# The Hidden Danger in Family Environment: The Role of Self-Reported Parenting Style in Cognitive and Affective Empathy Among Offenders

**DOI:** 10.3389/fpsyg.2021.588993

**Published:** 2021-02-04

**Authors:** Shaishai Wang, Huagang Hu, Xinyang Wang, Bo Dong, Tianyang Zhang

**Affiliations:** ^1^School of Public Health, Jiangsu Key Laboratory of Preventive and Translational Medicine for Geriatric Diseases, Medical College of Soochow University, Suzhou, China; ^2^School of Nursing, Medical College of Soochow University, Suzhou, China; ^3^Suzhou Prison, Suzhou, China; ^4^Department of Psychology, Suzhou University of Science and Technology, Suzhou, China; ^5^Research Center for Psychology and Behavioral Sciences, Soochow University, Suzhou, China

**Keywords:** parenting style, offenders, cognitive empathy, affective empathy, socioemotional well-being

## Abstract

Parenting styles are considered to have an important influence on the development of individuals and have been associated with empathy. The present study aimed to investigate the self-reported different parenting styles in childhood and adolescence and associated cognitive and affective empathy among offenders. Men incarcerated in prison in Jiangsu Province in China were invited to participate. Each consenting participant was asked to complete the Parental Bonding Instrument to collect information regarding the parenting styles they experienced in childhood and adolescence and the Interpersonal Reactivity Index to evaluate their empathy. A multivariable linear regression analysis was conducted to explore the associations between different parenting styles and the empathy of offenders, and a one-way multivariate analysis of variance and a *t*-test were used to explore the differences in cognitive and affective empathy with different degrees of parenting styles. The parental care and control factors in childhood and adolescence were significantly more strongly associated with empathy among offenders than the parental encouragement factor. There were different associations between the parental care and control factors and offenders’ empathy depending on whether the parenting styles were consistent or inconsistent. When the parenting styles were consistent, different degrees of parental care had a significant predictive effect on cognitive and affective empathy, while different degrees of parental control were only significantly associated with affective empathy among the offenders. When the parenting styles were inconsistent, different degrees of paternal and maternal control were associated with cognitive and affective empathy among the offenders. Our findings suggest that not only different parenting styles experienced in childhood and adolescence had different predictive effects on empathy among offenders but also the degrees of parenting styles and whether the paternal and maternal parenting styles were consistent or inconsistent may affect the patterns of parenting styles and empathy. Moreover, the parental control factor had a particular influence on empathy among the offenders. Our findings underscore the pressing need for adopting preventive monitoring measures or developing policies to improve parenting styles.

## Introduction

Dysfunction in the family of origin has consistently been found to be correlated with preadolescents’ antisocial behavior, and this dysfunction includes disruptions or omissions in the application of family management practices, broken families, institutional or foster home placements, low parental care, harsh discipline, physical and psychological abuse, parental antisocial and criminal behavior, parental mental health and substance use problems, delinquent siblings, and a large family size (Patterson and Stouthamer-Loeber, 1984; Ronis and Borduin, 2007; Gao et al., 2010; Lindberg et al., 2016). This dysfunction may cause elevated expression of psychopathic traits, which may lead to antisocial or violent behaviors (Lindberg et al., 2009). Parenting styles have proven to be a very important factor in the family of origin environment, and many previous studies have shown that among the factors related to the family of origin, parenting styles have the greatest influence on individuals (Lipps et al., 2013; Hillege et al., 2017; Musitu-Ferrer et al., 2019).

### Experienced Parenting Styles in Childhood and Adolescence

The presence of good parenting styles in the family of origin is very important for preventing juvenile delinquency (Kimonis et al., 2013). Currently, the main problems associated with the parenting styles of families are the degree of supervision and discipline provided by parents and whether parents can provide enough care and encouragement to their children (Musitu-Ferrer et al., 2019). Furthermore, many studies have shown that a positive parent-adolescent relationship protects adolescents against delinquency (Veen et al., 2011). These studies have shown that children and adolescents who often have conflicts with their parents or who receive limited parental support are at an increased risk of juvenile delinquency (Dekovic, 1999; Gorman-Smith et al., 2000). In addition, low levels of parental monitoring and high levels of harsh parental disciplining have been related to high levels of delinquent behavior in adolescents (Patterson and Stouthamer-Loeber, 1984; Lahey et al., 2008). Many previous studies have confirmed that several factors related to parental rearing patterns (i.e., caring, encouraging, and controlling) can affect individuals. Of these three factors, the control factor often attracts more attention (Llorca et al., 2017; Garcia and Serra, 2019). According to previous studies, the controlling factors in parenting styles may have profound influences on individuals (LeMoyne and Buchanan, 2011; Willoughby et al., 2015; Hosokawa et al., 2017). More negative parenting involving high levels of control has been found to be associated with more pessimistic views regarding marriage, higher levels of depression, and higher rates of prescribed medication for depression and anxiety (LeMoyne and Buchanan, 2011; Willoughby et al., 2015). More authoritarian parenting styles (e.g., overly strict and controlling) may involve an increased risk of child maltreatment (e.g., harsh verbal and physical disciplining practices) (Hosokawa et al., 2017). Many studies have also shown that a poor family of origin environment influences empathy among adults. The improper parenting of preadolescents is an important factor affecting empathy in adulthood (Cornell and Frick, 2007; Guo and Feng, 2017; Musitu-Ferrer et al., 2019). Meanwhile, some studies have noted that parenting styles may differentially influence cognitive and affective empathy, which further categorize empathy. These findings show that parenting styles are more likely to have a stronger association with affective empathy. Solantaus-Simula et al. (2002) reported that children with active empathy experienced more positive parenting, which was related to high prosocial behavior (Solantaus-Simula et al., 2002; Carlo et al., 2007; Stern et al., 2015).

### Cognitive and Affective Empathy Among Adult Offenders

Empathy is a multidimensional construct comprising cognitive, affective, and behavioral dimensions and has been advanced as a critical predictor of prosocial behavior and effectiveness in the workplace (Clark et al., 2019). Empathy allows individuals to share the affective states of others, predict others’ actions, and stimulate prosocial behavior. Recent evidence suggests that the following two systems are potentially responsible for empathy: a basic emotional contagion system and a more advanced cognitive perspective-taking system (Shamay-Tsoory et al., 2009). Recent studies have divided empathy into cognitive empathy and affective empathy based on the neural mechanisms or expression of empathy (Shamay-Tsoory et al., 2009). Cognitive empathy refers to the tendency to understand or the state of understanding others’ internal states (i.e., thoughts and affective states). Affective empathy or feeling the same affective state as another person is thought to be the phylogenetically earliest system of empathy (De Waal, 2008; Gonzalez-Liencres et al., 2013). Cognitive empathy is thought to develop later than affective empathy after children have acquired specific cognitive skills (De Waal, 2008; Shamay-Tsoory, 2011). People may show antisocial behavior and even criminal behavior when they lack empathy; thus, empathy deficits have been hypothesized to underlie the impairments in social interactions exhibited by those who engage in antisocial behavior (Van Zonneveld et al., 2017). Some previous studies have described the deficits in and characteristics of empathy among offenders; within the low and reactive aggression cluster, girls score higher on affective empathy, and compared with adults, a more consistent negative relationship exists between delinquency and affective empathy (but not cognitive empathy) in adolescents (Jolliffe and Farrington, 2004; Llorca et al., 2017). Empathy represents a potential mechanism that inhibits aggressive conduct and enhances prosocial behavior (Euler et al., 2017). Indeed, Jolliffe and Farrington (2004) found that cognitive empathy was more strongly negatively related to delinquency than affective empathy regardless of the type of offense or the age group studied (Jolliffe and Farrington, 2004). Explanations of criminal or antisocial behavior (both violent and/or sexual) have often hypothesized that a lack of empathy reduced the inhibition to cause harm to others, and empathy impairments have been related to aggressive, delinquent and antisocial behaviors (Proctor and Beail, 2007; Gery et al., 2009).

Previous studies have reported that many influencing factors are linked to empathy among offenders (Gery et al., 2009; Domes et al., 2013). Gery et al. (2009) supported the view that sex offenders might have impairments in the decoding of some emotional cues conveyed by conspecifics’ faces, which could have an impact on affective empathy. In addition, offenders with different levels of education could have different manifestations of cognitive empathy (Domes et al., 2013). However, attention has not been paid to the influence of parenting styles in the family of origin on offenders’ empathy. Many studies have found that parenting styles have a significant impact on the empathy of individuals (Guo and Feng, 2017; Garcia et al., 2018; Garcia and Serra, 2019). Guo and Feng (2017) noted that parenting styles are critical for fostering children’s empathy and prosociality (Guo and Feng, 2017). Several studies have found that exposure to different parenting styles during adolescence or young adulthood have different effects on individuals’ empathy (Garcia et al., 2018; Garcia and Serra, 2019). Offenders are often reported to have mood disorders or abnormal emotional development; among this special population, the patterns of association between parental rearing and empathy may be different (Smallbone et al., 2003; Llorca et al., 2017). Some studies have paid attention to the patterns of empathy associated with parenting rearing styles in a particular group of offenders (Kimonis et al., 2013; Llorca et al., 2017). Some results confirm that maternal warmth is positively associated with the empathic capacity in the offender community and that perceived parental support is an important factor promoting empathic concern, while perceived parental negative control could inhibit emotional development in the population of offenders. Previous studies have discussed the influences of control in parenting styles on empathy among offenders (Craissati et al., 2002; Rohner and Khaleque, 2003). One study found that paternal control tended to lead to social isolation and defectiveness and that high maternal control was predictive of impaired autonomy among offenders (Rohner and Khaleque, 2003). Craissati et al. (2002) supported the view that an affectionless control style of parental bonding was highly prevalent among sex offenders, and higher levels of parental control were noted among men with borderline personality disorder (Craissati et al., 2002). Many studies also found that parental control was significantly associated with lower rates of juvenile delinquency or produced only a small to moderate effect on reducing delinquency (Stattin and Kerr, 2000; Hoeve et al., 2009). Most offenders may have a poor family of origin environment and may not have experienced good parental rearing since preadolescence (Patterson and Stouthamer-Loeber, 1984; Lahey et al., 2008; Lindberg et al., 2016). Consequently, it is of the utmost importance to explore the associations between parenting styles in the family of origin and empathy among offenders.

Several studies have investigated the association between parenting styles and empathy among non-Chinese individuals (Carlo et al., 2007; Cornell and Frick, 2007; Stern et al., 2015). Many studies supported the view that many differences exist between Chinese and non-Chinese individuals in some aspects, such as social cognition, psychological state, family environment, etc. (Chang et al., 2001; Benner and Kim, 2009). Whether the results of studies in non-Chinese populations can be directly generalized to the Chinese population remains uncertain. Studies focusing on the association between experienced parenting styles and empathy among offenders in China are rare. A better understanding of parenting styles and associated risk factors among this special population could draw more attention from all circles in society, which is conducive to the prevention of more violent behavior in adulthood by improving the experienced parenting styles of adolescents in the family of origin.

We hypothesize that different parenting styles in childhood and adolescence could have different predictive effects on empathy, strict parenting styles may have a more significant effect, and different patterns of parenting styles may also have different associations with empathy. To test this hypothesis, we administered the abovementioned scales assessing parenting styles and empathy to offenders to obtain information regarding their experienced parenting style and their capacity for empathy. The current study analyzed offenders’ demographic information and other psychological information. Based on this hypothesis, we predicted that parenting styles may be associated with affective empathy and cognitive empathy among offenders (Carlo et al., 2007; Shamay-Tsoory et al., 2009). Cognitive empathy and affective empathy among offenders may have different associations with parenting styles when paternal and maternal parenting styles are inconsistent (Solantaus-Simula et al., 2002; Guo and Feng, 2017; Garcia and Serra, 2019).

## Methods

### Participants

This study is based on data from offenders recruited from a prison in Jiangsu Province. In this project, Soochow University coordinated a collaboration among a prison in Jiangsu Province, the Center for Disease Control and Prevention (CDC) of Xiangcheng District in Suzhou and Soochow University. The participants were offenders who disrupted social order or engaged in violent behaviors. In total, 994 prisoners were selected because their fathers and mothers were their caregivers. The age of the participants ranged from 17 to 67 years (mean age = 39.93, SD = 8.76). In total, 26.1% of the offenders were the only child in their families. Of the total sample, 37.9% were unmarried, 27.8% were divorced, 1.2% were widowers, and 32.9% were married. Among the participants, 13.6% did not graduate from junior high school, most participants (62.5%) reported having a junior high school diploma, 17.2% indicated having a high school diploma, and 6.5% had a college degree. All survey data were collected after informed consent was obtained from the participants. The records of the offenders were provided by the prison from the Jiangsu Province database according to a data sharing agreement. The presented research was approved by Soochow University’s institutional review boards for research conducted with human subjects.

All participants were recruited from a prison in Jiangsu Province after successful negotiation and establishment of an arrangement by the Suzhou prison, CDC and Soochow University. Most offenders cooperated with our investigations and were accompanied by the prison’s police.

### Procedures

In total, 1324 offenders admitted to the counseling room were screened for inclusion. The participants’ data were collected using different scales as follows: 1324 offenders aged between 17 and 67 years completed scales with questions regarding their social demographics, parenting styles, trauma, empathy and other characteristics in the counseling room. All 1324 returned a completed scale, of which three responses were invalid and were excluded from the subsequent data analysis. Our study used only the scales assessing parenting styles and empathy, and the survey was designed according to the purposes of our study. The scales are available from the authors upon request.

### Measures

#### Parenting Styles

The parenting style questionnaire is a self-report scale used to assess individuals’ cognition of the parenting styles they experienced in childhood and adolescence (before the age of 16 years) (Parker et al., 1979). Parenting styles refer to the relatively stable behavior styles of parents in raising and educating their children. According to attachment theory, parental care, namely, love, gentleness, closeness, and low control, are necessary for children’s safe attachment and normal development. Parental control, namely, intervention, obedience, overprotection, and low care, contributes to children’s insecure attachment mode and subsequent psychological disorders. The questionnaire was divided into the maternal version (Parental Bonding Instrument-Mother; PBI-M) and the paternal (i.e., father) version (PBI-F) with 23 items each and used to assess the following three factors: caring, encouraging autonomy and controlling. The following 4-point Likert scoring was applied: “0” represented “does not conform very well”, “1” represented “does not conform”, “2” represented “match”, and “3” represented “fits”. The Cronbach’s alpha coefficients of PBI-F and PBI-M in this study were 0.821 and 0.776, respectively.

#### Empathy

To assess empathy in a multidimensional manner, we administered the Interpersonal Reactivity Index (IRI). The IRI (Davis, 1983) is a 22-item self-report questionnaire that measures two components of empathy (Davis, 1983). To date, the IRI is the only published measure that allows a multidimensional assessment of empathy. Empathy in the broadest sense refers to the reactions of an individual to the observed experience of another individual. The IRI describes four separate aspects of empathy, which are assessed in relation to measures of social functioning, self-esteem, emotional functioning, and sensitivity to others. Two subscales, namely, perspective taking (PT) and fantasy (FS), measure cognitive empathy (CE), while the other two subscales, namely, empathic concern (EC) and personal distress (PD), measure affective empathy (AE). More specifically, the PT subscale measures the tendency of individuals to cognitively place themselves in the position of others, thereby adopting their psychological viewpoint, while the FS subscale provides an indication of the extent to which people can immerse themselves in and identify with the feelings and actions of fictitious characters. The EC subscale is designed to measure the capacity to experience feelings of compassion, warmth, and concern in response to other people, whereas the PD subscale evaluates subjective feelings of unease and discomfort in reaction to observing anguish and pain endured by others (Smallbone et al., 2003). The Cronbach’s alpha coefficient of the IRI in this study was 0.862.

### Data Analysis

First, a descriptive analysis was performed to examine the psychological state of the offenders with different ages and levels of education. A multivariable linear regression was used to identify the influence of the three different factors of parenting styles on empathy. Then, the three different parenting styles were divided into the following three degrees: high (top 27% of the score), medium (excluding high and low scores) and low (bottom 27% of the score). To investigate whether different levels of parenting styles impact empathy among individuals, we conducted a series of one-way multivariate analyses of variance (MANOVAs). To observe whether an individual’s empathy is differentially affected when the father and mother show different levels of the same parenting style factor, the parenting performance of the father and mother in a specific parenting style factor was divided into the following two types: father-high and mother-low (FHML) and father-low and mother-high (FLMH). Then, a t-test was used to determine whether the difference between the paternal and maternal parenting styles affected the individuals’ empathy.

## Results

### Mental Health Problems and Parenting Style Characteristics of the Participants

In total, 994 male offenders with valid data were included in the present study. We describe the psychological states, such as anxiety, trauma, etc., of the offenders with different ages and education levels in Table 1. Meanwhile, the three different factors of the paternal and maternal parenting styles and the four items representing empathy based on the parenting styles of the parents are also expressed (mean and SD) across ages and education levels in Table 1 (*n* = 994). Overall, there were no significant differences in the parenting styles and the four empathy items among the different age groups. With improvements in the education level, the degree of parental care and encouragement and the EC (*F*_(994)_ = 8.05, *p* < 0.01), PT (*F*_(994)_ = 6.27, *p* < 0.01) and FS (*F*_(994)_ = 5.50, *p* < 0.01) empathy items showed an increasing trend, while the degree of parental control and PD (*F*_(994)_ = 2.98, *p* < 0.05) showed a decreasing trend.

**TABLE 1:** Characteristics of mental health problems among offenders by demographic factors (*n* = 994).

	Age				Education			
		
	< 30 (10.7%)	30-39(40.5%)	40-49(33.1%)	≥ 50 (15.4%)	Primary school and below (13.6%)	Junior high school (62.5%)	High school (17.2%)	College and above (6.5%)
Maternal care	22.76(5.31)	22.72 (4.32)	22.47 (4.67)	22.53 (4.19)	21.54 (4.56)	22.44 (4.40)	23.59 (4.63)	23.96 (4.73)
Maternal encouragement	10.94(4.16)	10.67 (3.84)	10.75 (4.01)	11.12 (3.49)	9.85 (4.26)	10.83 (3.80)	11.22 (3.78)	11.33 (3.86)
Maternal control	6.25(2.41)	5.82 (2.42)	5.69 (2.42)	5.60 (2.50)	5.91 (2.74)	5.86 (2.33)	5.64 (2.48)	5.25 (2.57)
Paternal care	19.54(4.73)	19.60 (4.28)	19.24 (4.54)	19.45 (4.26)	18.43 (4.77)	19.31 (4.25)	20.42 (4.40)	20.41 (4.53)
Paternal encouragement	11.48(3.85)	11.47 (3.50)	11.18 (3.62)	11.32 (3.32)	10.23 (3.87)	11.39 (3.43)	11.75 (3.52)	12.29 (3.55)
Paternal control	6.56(2.66)	5.86 (2.57)	5.91 (2.48)	5.91 (2.42)	6.07 (2.58)	6.10 (2.41)	5.64 (2.65)	5.24 (3.02)
Perspective taking	8.43(4.07)	8.20 (3.90)	7.83 (3.82)	8.11 (3.65)	7.79 (4.07)	7.82 (3.65)	8.74 (4.22)	9.59 (3.92)
Personal distress	5.23(3.98)	4.97 (4.05)	5.24 (3.98)	5.03 (3.51)	5.71 (3.79)	5.18 (3.99)	4.51 (3.72)	4.50 (4.10)
Empathic concern	16.36(3.35)	16.39 (3.48)	16.42 (3.56)	16.41 (3.43)	15.48 (3.52)	16.30 (3.39)	17.01 (3.67)	17.66 (3.18)
Fantasy	13.57(4.07)	12.38 (3.87)	12.48 (3.61)	12.53 (3.61)	11.93 (3.81)	12.40 (3.54)	13.25 (4.31)	13.69 (4.08)
State anxiety	46.35 (10.13)	46.48 (10.19)	46.64 (10.05)	45.29 (9.03)	48.17 (9.21)	46.84 (9.72)	44.66 (10.25)	42.05 (11.39)
Trait anxiety	47.45 (9.29)	46.82 (9.39)	46.94 (9.09)	45.85 (8.29)	48.85 (8.87)	47.03 (8.90)	45.60 (9.31)	43.14 (9.83)
Cumulated number of trauma (before the age of 18)	2.16 (2.89)	1.77 (2.45)	1.32 (1.84)	1.28 (2.02)	1.55 (2.06)	1.64 (2.26)	1.49 (2.58)	1.39 (1.87)
Cumulated number of trauma (after the age of 18)	1.90 (2.24)	2.34 (2.58)	2.45 (2.60)	2.35 (2.90)	2.67 (2.74)	2.30 (2.55)	2.40 (2.86)	1.74 (2.01)

### Multivariable Logistic Regression Analysis of Empathy Across Parenting Styles

Multiple linear regression analyses were performed to determine the associations between different parenting styles and different items on the empathy scale. Experience with paternal and maternal caring was associated with an increasing trend in EC (all *p* for trend < 0.05), while experience with paternal control was associated with a decreasing trend in EC (*p* for trend < 0.05). All parenting styles other than paternal encouragement were associated with increasing trends in PT (all *p* for trend < 0.05). Experience with paternal and maternal control was associated with an increasing trend in PD (all *p* for trend < 0.001). Experience with paternal and maternal care and maternal control were associated with an increasing trend in FS (all *p* for trend < 0.05) (see Table 2).

**TABLE 2:** Multivariable linear regression analysis of empathy with parenting styles (*n* = 994).

Variable	Constant term	*B*	Standard error	*Beta*	*t*	*p*
EC	Constant term	10.28	0.70		14.61	< 0.001
	Maternal care	0.24	0.03	0.31	7.68	< 0.001
	Maternal encouragement	–0.007	0.03	–0.008	0.21	0.83
	Maternal control	–0.06	0.05	–0.04	1.26	0.2
	Paternal care	0.07	0.03	0.09	2	< 0.05
	Paternal encouragement	0.01	0.04	0.01	0.37	0.71
	Paternal control	–0.09	0.05	–0.07	1.99	< 0.05
PT	Constant term	–0.43	0.8		0.54	0.58
	Maternal care	0.11	0.03	0.13	3.04	< 0.01
	Maternal encouragement	0.11	0.04	0.11	2.61	< 0.01
	Maternal control	0.17	0.05	0.11	3.09	< 0.01
	Paternal care	0.08	0.04	0.1	2.14	< 0.05
	Paternal encouragement	0.10	0.05	0.09	1.88	0.06
	Paternal control	0.14	0.05	0.09	2.55	< 0.05
PD	Constant term	2.78	0.82		3.35	< 0.001
	Maternal care	–0.04	0.03	–0.04	1.09	0.27
	Maternal encouragement	0.03	0.04	0.03	0.86	0.38
	Maternal control	0.28	0.05	0.17	4.8	< 0.001
	Paternal care	–0.07	0.04	–0.08	1.73	0.08
	Paternal encouragement	0.07	0.05	0.06	1.27	0.2
	Paternal control	0.30	0.05	0.19	5.24	< 0.001
FS	Constant term	6.62	0.82		8.04	< 0.001
	Maternal care	0.14	0.03	0.17	4.01	< 0.001
	Maternal encouragement	–0.06	0.04	–0.06	1.55	0.12
	Maternal control	0.20	0.05	0.13	3.45	< 0.001
	Paternal care	0.10	0.04	0.12	2.43	< 0.05
	Paternal encouragement	–0.009	0.05	–0.008	0.16	0.87
	Paternal control	0.03	0.05	0.02	0.56	0.57

*EC represents empathic concern; PT represents perspective taking; PD represents personal distress; and FS represents fantasy.*

### Analysis of Variance of Empathy Across Different Paternal and Maternal Parenting Styles

According to the results of the regression analysis, the three different parenting styles of the father and mother had different effects on the individuals’ empathy. According to the scores of each parenting style factor, the three parenting styles were divided into three groups, i.e., high, medium, and low, as described above to indicate the different degrees of each parenting style factor. An analysis of variance (ANOVA) was used to compare the differences in the four items of empathy based on the three parenting styles across different degrees of the father and mother.

Follow-up univariate ANOVAs of each variable were conducted separately and revealed that there were statistically significant differences in four different empathy items among individuals exposed to different degrees of the three paternal and maternal parenting styles. The group that experienced high parental care and encouragement had significantly higher EC, PT, and FS scores and had significantly lower PD scores than the group that experienced low levels of parental care and encouragement. The group that experienced higher levels of parental control had significantly lower EC scores but significantly higher PD scores than the group that experienced low levels of parental control. When the parental control level was high, the PT score was the highest, and those who experienced medium levels of parental control had significantly lower PT scores than those who experienced low levels of parental control. When the paternal control level was low, the FS scores were the highest, and the group that experienced high levels of paternal control had significantly lower FS scores than the group that experienced medium levels of paternal control; when the level of maternal control was high, the FS scores were the highest, and the group that experienced medium levels of maternal control had significantly lower FS scores than the group that experienced low levels of maternal control (see Tables 3,4).

**TABLE 3:** Empathy differences under different degrees of parental care (*n* = 994).

Group	*n*	EC		PT		PD		FS	
					
		Father	Mother	Father	Mother	Father	Mother	Father	Mother
High	268	18.09 ± 3.38	18.18 ± 3.16	9.71 ± 4.42	9.43 ± 4.11	4.60 ± 4.02	4.58 ± 3.90	13.72 ± 4.63	13.39 ± 4.32
Medium	457	16.17 ± 3.24	16.44 ± 3.16	7.81 ± 3.16	7.85 ± 3.57	5.10 ± 3.67	4.75 ± 3.71	12.23 ± 3.28	12.49 ± 3.68
Low	269	15.12 ± 3.33	14.55 ± 3.36	6.95 ± 3.82	7.15 ± 3.72	5.59 ± 4.25	6.20 ± 4.15	12.00 ± 3.39	11.87 ± 3.19
	***F***	56.18	85.51	39.4	26.2	4.26	15.02	17.85	11.14
	***p***	< 0.001	< 0.001	< 0.001	<0.001	< 0.05	< 0.001	< 0.001	< 0.001

*EC represents empathic concern; PT represents perspective taking; PD represents personal distress; and FS represents fantasy.*

**TABLE 4:** Empathy differences under different degrees of parental control (*n* = 994).

Group	*n*	EC		PT		PD		FS	
					
		Father	Mother	Father	Mother	Father	Mother	Father	Mother
High	268	15.27 ± 3.41	15.34 ± 3.47	8.60 ± 3.81	8.73 ± 4.00	6.79 ± 4.17	6.74 ± 4.13	12.85 ± 3.69	12.39 ± 3.95
Medium	457	16.51 ± 3.41	16.52 ± 3.36	7.67 ± 3.57	7.60 ± 3.49	4.76 ± 3.57	4.89 ± 3.76	12.09 ± 3.39	12.25 ± 3.57
Low	269	17.33 ± 3.37	17.25 ± 3.43	8.29 ± 4.28	8.27 ± 4.20	3.97 ± 3.77	3.82 ± 3.48	13.10 ± 4.37	12.74 ± 3.93
	***F***	25.02	21.58	5.43	7.86	4.26	41.2	7.14	3.19
	***p***	< 0.001	< 0.001	0.004	< 0.001	< 0.05	< 0.001	< 0.001	< 0.05

*EC represents empathic concern; PT represents perspective taking; PD represents personal distress; FS represents fantasy.*

We noted some differences in the results between the one-way multivariate ANOVA and multivariable linear regression analyses in the current study, and we explain these results from the perspective of the statistical analyses. The results of the multiple linear regression analysis are not completely consistent with the results of the ANOVA. The results of the multiple regression analysis show that parental care can predict three aspects of empathy (PT, FS and EC), while the ANOVA revealed that parental care influenced all four aspects of empathy. The regression analyses showed that the degree of paternal control could predict three aspects of empathy (PT, EC and PD), and maternal control could predict three different aspects of empathy (PT, FS and FS), whereas the ANOVA showed that parental control influenced all four aspects of empathy (see Tables 2, 3). We speculate that this inconsistency may be due to the indirect effects of parental care on PD, suggesting that maternal care for personal distress may not have a direct effect, while the other factors associated with parenting styles, such as maternal and paternal control, may have an impact on personal distress. However, there is collinearity between maternal care and these two factors; thus, in the single factor analysis of variance, paternal care is also the result of the significant differences, and there was no predictive effect of parental care on PD after adjusting for some factors that exhibit collinearity with maternal care in the multivariate regression. Similarly, paternal control has an indirect effect on FS, and maternal control has an indirect effect on EC (Nakamura et al., 2011).

### Analysis of Variance and *t*-Test of Empathy Based on Consistent and Inconsistent Parenting Styles

After examining the parenting styles of fathers and mothers separately, we also examined whether the individuals’ four empathy items differed when the degree of the father and mother parenting styles were consistent or inconsistent. When the parenting styles were consistent, the three parenting styles were divided into three groups based on the scores, i.e., high, medium and low, using the above classification criteria. The inconsistent parenting styles were categorized into the following two types: FHML (top 27% of the score of the father and bottom 27% of the score of the mother) and FLMH (bottom 27% of the score of the father and top 27% of the score of the mother). The MANOVA results indicated that there were statistically significant differences in the four different empathy items among individuals who experienced different degrees of paternal and maternal parenting styles when the parenting styles were consistent, and the t-test results revealed that there were statistically significant differences in the four empathy items among individuals who experienced different degrees of paternal and maternal parenting styles when the parenting styles were inconsistent.

**FIGURE 1:**
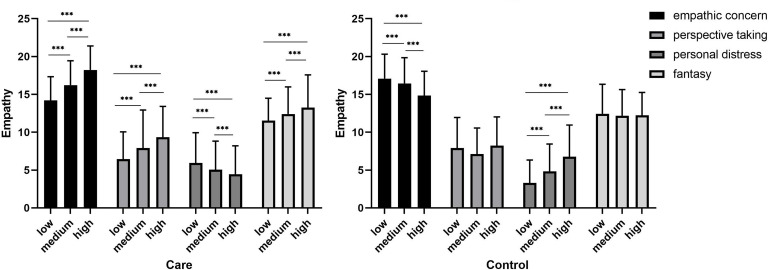
Empathy differences under different degrees of parental care and control when the paternal and maternal parenting styles are consistent (*n*(care) = 963, *n*(control) = 919). ^∗∗∗^ *p* < 0.001.

**FIGURE 2:**
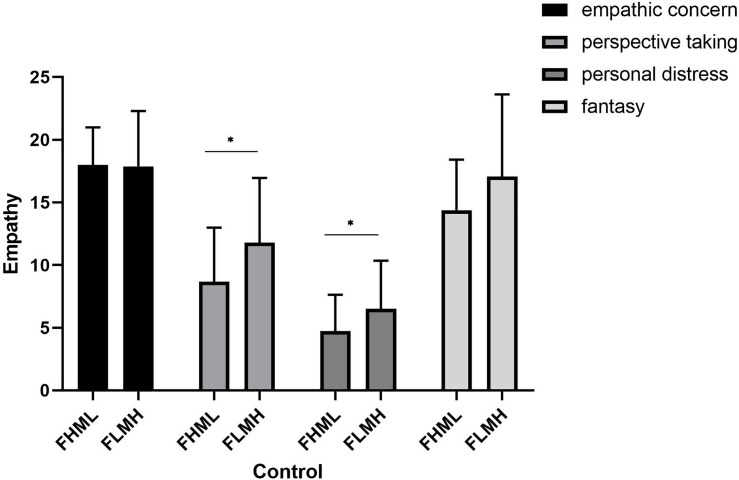
Empathy differences under different degrees of paternal and maternal control when the paternal and maternal parenting styles are inconsistent (*n* = 59). FHML, father-high and mother-low; FLMH, father-low and mother-high. ^∗^*p* < 0.05.

When the paternal and maternal parenting styles were consistent, the group that experienced high parental care had significantly higher EC (*F*_(963)_ = 81.44, *p* < 0.001), PT (*F*_(963)_ = 32.49, *p* < 0.001) and FS (*F*_(963)_ = 11.34, *p* < 0.001) scores but significantly lower PD scores (*F*_(963)_ = 7.95, *p* < 0.001) than the group that experienced low parental care. The group that experienced high parental encouragement had significantly higher EC (*F*_(924)_ = 36.32, *p* < 0.001), PT (*F*_(924)_ = 37.11, *p* < 0.001) and FS (*F*_(924)_ = 9.57, *p* < 0.001) scores than the group that experienced lower parental encouragement, and there were no significant differences in PD. The group that experienced high parental control had significantly lower EC scores (*F*_(919)_ = 2.66, *p* < 0.001) but significantly higher PD scores (*F*_(919)_ = 43.8, *p* < 0.001) than the group that experienced lower parental control. When the paternal and maternal parenting styles were inconsistent, the proportion of participants that experienced FLMH and FHML care was too small to perform a meaningful statistical comparison; thus, there is no further discussion regarding this group in the subsequent analysis. The group that experienced FHML encouragement had significantly lower PD scores (*t*_(50)_ = 2.39, *p* = 0.02) than the group that experienced FLMH encouragement, and there were no significant differences in the EC, PT and FS scores. The group that experienced FHML control had significantly lower PT (*t*_(59)_ = 2.52, *p* < 0.05) and PD (*t*_(59)_ = 2.01, *p* < 0.05) scores than the group that experienced FLMH control, and there were no significant differences in the EC and FS scores (see Figures 1,2).

## Discussion

In the present study, we found that parental care and control in childhood and adolescence had a significantly greater influence on the empathy of offenders than parental encouragement. In addition, regarding paternal and maternal parenting styles, the predictive model of the impact of parental care on the empathy of offenders had similar patterns, but there were differences in the predictive patterns of the impact of paternal and maternal parental control on the empathy of offenders. Moreover, the present study revealed that there were different associations between the parental care and control and offenders’ empathy depending on whether the parenting styles were consistent or inconsistent, and the most interesting result was that parental control had a special influence on the empathy of offenders. Although the results of a single study are far from conclusive, our study offers new avenues for exploring and understanding the associations between different parenting styles and empathy of individuals.

Our hypothesis was based on the fact that empathy was usually divided into cognitive empathy and affective empathy in previous studies (Shamay-Tsoory et al., 2009; Shamay-Tsoory, 2011). Perspective taking and fantasy were used to measure cognitive empathy, and empathic concern and personal distress were used to measure affective empathy (De Waal, 2008; Shamay-Tsoory, 2011; Gonzalez-Liencres et al., 2013). Based on the regression analyses, the present study showed that the three different parenting styles in childhood and adolescence had different influences on the cognitive and affective empathy of offenders. On the one hand, paternal and maternal care had a significant influence on the individuals’ cognitive empathy, including perspective taking and fantasy, but a weaker influence on affective empathy, i.e., only influenced empathic concern. On the other hand, maternal control had a significant influence on the individuals’ cognitive empathy, including perspective taking and fantasy, while paternal control had a significant influence on affective empathy, including empathic concern and personal distress, among the offenders. Moreover, only maternal encouragement had a significant predictive influence on the perspective taking of offenders. Several previous studies revealed that parental care and control have significant influences on empathy of individuals, but less attention has been paid to parental encouragement (Reti et al., 2002; Lyons et al., 2016). However, several studies have found that parental encouragement has no significant influence on the empathy of individuals (Rohner and Khaleque, 2003; Garcia and Serra, 2019). Based on the regression results, the parental care and control factors had a more significant impact on the individuals’ empathy than the encouragement factor. Compared with offenders, normal participants may experience gentler parenting styles usually characterized by more care and encouragement and less control; thus, such parenting styles may be good promoters of individuals’ emotional and empathic development. According to previous research, caring generally leads to warmth, while control often leads to harm (Solantaus-Simula et al., 2002; Parlar et al., 2014; Llorca et al., 2017). Therefore, we pay more attention to the associations between the parental care and control factors and offenders’ ability to empathize in the subsequent discussion.

To further examine the associations between the parental care and control factors in childhood and adolescence and the empathy of offenders, we divided parental care and control into three groups, namely, high, medium and low, to compare the differences in the four empathy items with the three different degrees of parenting styles. In addition, the ANOVA showed that there were significant differences in cognitive and affective empathy with different degrees of paternal and maternal care. High levels of parental care had a positive predictive effect on cognitive empathy (i.e., perspective taking and fantasy) compared with low levels of parental care. There was also a significant influence on affective empathy, which was characterized by higher empathic concern and lower personal distress. Some studies suggested that parental care may be positively associated with perspective taking and empathic concern, and the results of this study are consistent with this conclusion (Reti et al., 2002; Britton and Fuendeling, 2005). One study found that paternal care influenced affective empathy and that maternal care was related to cognitive empathy in men, while none of the parental care variables were related to cognitive empathy in women (Lyons et al., 2016). This study revealed that both paternal and maternal care influenced affective and cognitive empathy among male offenders. It may be that most offenders experienced a poor family of origin environment in childhood and adolescence and that these individuals’ empathy is more sensitive to parental care (Cornell and Frick, 2007; Guo and Feng, 2017; Musitu-Ferrer et al., 2019). There were also significant differences in cognitive and affective empathy associated with different degrees of paternal and maternal control. Parental control had a significant influence on affective empathy with a negative predictive effect on empathic concern and a positive predictive effect on personal distress. In many previous studies, parental control during adolescence has been proven to have a negative predictive effect on individuals’ empathic concern; in addition, one study revealed that parental control was not significantly associated with the empathic concerns of individuals, although perceived parental control had a significant predictive effect on individuals’ empathic concern (Asano et al., 2016). The ANOVA results showed that higher and lower levels of parental control had a positive influence on cognitive empathy and that medium levels of parental control had a negative predictive effect on cognitive empathy. Asano et al. (2016) revealed that parental control and perceived parental control during early adolescence directly increased perspective taking (Asano et al., 2016). Mcelroy and Rodriguez (2008) reported that parental control in childhood was significantly negatively associated with perspective taking (Mcelroy and Rodriguez, 2008). Our study revealed that the degree of parental control is very important and that either high or low levels of parental control may promote the development of offenders’ cognitive empathy; high levels of parental control may have a greater promoting effect.

The growth of most individuals in the family of origin environment is influenced by both fathers and mothers; however, previous studies paid limited attention to the influences on the empathy of individuals when the paternal and maternal parenting styles are consistent or inconsistent in the family of origin (Solantaus-Simula et al., 2002; Guo and Feng, 2017; Garcia and Serra, 2019). Notably, some participants report that their main caregivers were their grandparents or that their grandparents also played a very important role in their upbringing. The influence of such parenting styles on individual development has also been mentioned in previous studies (Nanthamongkolchai et al., 2011). The findings suggest that the impact of parental rearing on individuals’ psychological and emotional development is likely greater than that of the parenting patterns of grandparents because child development in those who were reared by parents was affected only by the child rearing factor, while the factors affecting the development of children reared by grandparents included both the level of the family income and the child rearing factor. The present study also divided the paternal and maternal care and control factors into three degrees, i.e., high, medium and low, to observe whether there were significant differences in the empathy of offenders when the paternal and maternal parenting styles were consistent or inconsistent. When the paternal and maternal parenting styles were consistent, parental care had a positive predictive effect on cognitive empathy. There were also significant differences in affective empathy, which manifested as higher empathic concern and lower fantasy. The theory is consistent with the pattern of the influence of paternal and maternal care on offenders’ empathy revealed earlier in this study. The paternal and maternal care factors, separately or together, had an influence on the offenders’ cognitive and affective empathy, and the same predictive trends were observed in both types of empathy. In some previous studies, parental care refers to a warm parenting style and has been revealed to have a positive effect on the empathy of individuals (Parlar et al., 2014; Llorca et al., 2017). Parlar et al. (2014) reported that higher levels of paternal care on the PBI were predictive of higher scores on the perspective taking subscale of the IRI (Parlar et al., 2014). The indulgent parenting style, which mainly involves parental care, has been proven to have a significant predictive effect on higher empathy, but the study did not reveal the specific influences on cognitive and affective empathy (Llorca et al., 2017). Parental control had a significant influence on affective empathy, but not cognitive empathy, among the offenders. Empathic concern and personal distress showed opposite trends with different degrees of parental control, and this pattern was consistent with the trends associated with paternal and maternal control. When the paternal and maternal parenting styles were inconsistent, perspective taking and personal distress significantly differed based on the different levels of paternal and maternal control. Thus, parental control had a significant predictive effect on the cognitive and affective empathy of the offenders with a larger effect on cognitive empathy. Furthermore, the results revealed that paternal and maternal control had different influencing patterns on offenders’ empathy when the parenting styles were consistent or inconsistent. Parental control usually refers to strictness or an authoritarian parenting style and has been found to be associated with the empathy of individuals (Craissati et al., 2002; Rohner and Khaleque, 2003). However, few studies have paid attention to the influences on cognitive and affective empathy; meanwhile, these studies did not discuss the effects on empathy when the degree of paternal and maternal control was consistent or inconsistent. Notably, parenting styles consist of paternal and maternal rearing styles, and previous studies have supported the notion that fathers and mothers may have different rearing attitudes regarding the development of individuals (Dekovic, 1999; Solantaus-Simula et al., 2002; Gao et al., 2010). Furthermore, the family of origin environment in particular populations (e.g., a group of offenders) is generally dysfunctional, and family members, including the father and mother, may have exhibited certain negative habits or antisocial behavior or have a criminal history (Lindberg et al., 2009), which may lead to different parenting attitudes and styles of parenting during periods of growth (e.g., childhood and adolescence). The paternal and maternal care and control factors may have differential influences on empathy among offenders during these developmental periods, and the effects of inconsistent or consistent patterns of paternal and maternal parenting styles on offenders’ empathy are different.

Previous studies have investigated the association between parenting styles and empathic ability in the general population, and the results show that a positive approach to rearing could have a greater predictive effect on the development of children’s empathy ability and that lower father care may be negatively associated with empathy (Solantaus-Simula et al., 2002; Parlar et al., 2014); thus, the positive factors (e.g., parental care) of parenting styles may have a positive predictive effect on the empathy ability of normal participants, while negative factors (e.g., parental control) may have a more significant negative association in the particular population of offenders.

## Limitations and Future Directions

The present study is among the few studies exploring the associations between different degrees of parenting styles experienced in childhood and adolescence and cognitive and affective empathy among offenders. Our finding that different paternal and maternal styles have different predictive effects on the empathy of offenders is interesting, and the results revealed that parental care and control had a more significant influence on individuals’ empathy than parental encouragement. Previous studies have paid more attention to the influences of overall parenting styles on individuals and did not examine the influence of the three parenting styles (Lipps et al., 2013; Hillege et al., 2017; Musitu-Ferrer et al., 2019). The most important finding was that different degrees of the paternal and maternal care and control had different associations with cognitive and affective empathy among offenders. Based on whether the paternal and maternal parenting styles were consistent or inconsistent, parental control also had different predictive effects on cognitive and affective empathy among offenders.

However, our study also has some limitations. First, the study sample in the present study was limited to male offenders, and whether or how gender may impact empathy is unclear. Some studies have supported the view that women are more empathic than men, and our future research aims to recruit subjects of different genders (Toussaint and Webb, 2005; Mestre et al., 2009). Furthermore, the information regarding the parenting styles experienced by the offenders was mainly derived from the questionnaire, which relied on the recall of parental rearing before the age of 16 years. There may have been recall bias, and the information collected in the present study may not be sufficient to fully characterize the experienced parental rearing patterns. A longitudinal study is needed to establish the impact of different paternal and maternal rearing styles on the development of empathy. Finally, this association needs to be further studied in other populations, such as students at different stages. These associations were investigated in a specific population of prison inmates because the participants in this study were more likely to have deficits in empathy and have experienced poor parental rearing styles in their family of origin; the associations between different degrees of the parental styles and cognitive and affective empathy may differ from those in the general population (Patterson and Stouthamer-Loeber, 1984; Gilligan and Lee, 2005).

In conclusion, the current study shows that there were different associations between different parenting styles in childhood and adolescence and cognitive and affective empathy. The results revealed that different degrees of parenting styles had different predictive effects on offenders’ cognitive and affective empathy depending on whether the paternal and maternal parenting styles were consistent or inconsistent. In addition, parental control had particular influences on the cognitive and affective empathy of offenders, highlighting the pressing need for paying more attention to the control of parental rearing. These findings may provide preliminary empirical evidence to improve and enhance the capacity for cognitive and affective empathy from the perspective of improving parental rearing styles. These associations need to be confirmed and replicated in other populations, and well-designed longitudinal cohort studies are needed to investigate the longitudinal effects of parenting styles on the development of empathy in the general population.

## Data Availability Statement

The raw data supporting the conclusions of this article will be made available by the authors, without undue reservation.

## Ethics Statement

The studies involving human participants were reviewed and approved by Health Development Research Center of Soochow University. The participants provided their written informed consent to participate in this study.

## Author Contributions

TZ proposed the main research idea. TZ and BD made the research design. HH and XW conducted the investigation. SW and HH ran the statistics. SW, BD, and TZ made the discussion and wrote the manuscript. All authors contributed to the article and approved the submitted version.

## Conflict of Interest

The authors declare that the research was conducted in the absence of any commercial or financial relationships that could be construed as a potential conflict of interest.
